# Pathway-based analysis of anthocyanin diversity in diploid potato

**DOI:** 10.1371/journal.pone.0250861

**Published:** 2021-04-29

**Authors:** Maria Angelica Parra-Galindo, Johana Carolina Soto-Sedano, Teresa Mosquera-Vásquez, Federico Roda

**Affiliations:** 1 Facultad de Ciencias Agrarias, Universidad Nacional de Colombia, Sede Bogotá, Bogotá, Colombia; 2 Departamento de Biología, Facultad de Ciencias, Universidad Nacional de Colombia, Sede Bogotá, Bogotá, Colombia; 3 Max Planck Tandem Group, Facultad de Ciencias, Universidad Nacional de Colombia, Sede Bogotá, Bogotá, Colombia; Agriculture and Agri-Food Canada, CANADA

## Abstract

Anthocyanin biosynthesis is one of the most studied pathways in plants due to the important ecological role played by these compounds and the potential health benefits of anthocyanin consumption. Given the interest in identifying new genetic factors underlying anthocyanin content we studied a diverse collection of diploid potatoes by combining a genome-wide association study and pathway-based analyses. By using an expanded SNP dataset, we identified candidate genes that had not been associated with anthocyanin variation in potatoes, namely a Myb transcription factor, a Leucoanthocyanidin dioxygenase gene and a vacuolar membrane protein. Importantly, a genomic region in chromosome 10 harbored the SNPs with strongest associations with anthocyanin content in GWAS. Some of these SNPs were associated with multiple anthocyanin compounds and therefore could underline the existence of pleiotropic genes or anthocyanin biosynthetic clusters. We identified multiple anthocyanin homologs in this genomic region, including four transcription factors and five enzymes that could be governing anthocyanin variation. For instance, a SNP linked to the phenylalanine ammonia-lyase gene, encoding the first enzyme in the phenylpropanoid biosynthetic pathway, was associated with all of the five anthocyanins measured. Finally, we combined a pathway analysis and GWAS of other agronomic traits to identify pathways related to anthocyanin biosynthesis in potatoes. We found that methionine metabolism and the production of sugars and hydroxycinnamic acids are genetically correlated to anthocyanin biosynthesis. The results contribute to the understanding of anthocyanins regulation in potatoes and can be used in future breeding programs focused on nutraceutical food.

## Introduction

Potato (*Solanum tuberosum* L.) is the main non-cereal food consumed worldwide [1] and the vegetable with the highest antioxidant contribution to human diet [2]. Within the *S*. *tuberosum* L. species, the Group Phureja is composed of diploid potatoes (2n = 2x = 24) with short-day adaptation and a lack of tuber dormancy that are widely grown by local farmers in the Andes mountains range of South America. Landraces from the Andes were the first domesticated potatoes and the main origin of cultivars, developed after the colonization of America and grown in most of the rest of the world today [3]. There is growing interest in recovering genetic variation for agronomic traits, one of these traits is the presence of bioactive compounds that are present in landraces and was lost during the improvement of cultivars [4, 5]. One of the bioactive compounds with increasing interest is reflected in the red and purple coloration in the skin and flesh of potato tubers [6–8], which result from the accumulation of anthocyanin pigments [9, 10]. Multiple potential health benefits have been described to the consumption of anthocyanin-pigmented potatoes, including the protection against several diseases, mainly because of their antioxidant capacity [11–13]. Phureja potatoes present a particularly broad variation in anthocyanin contents [14], with total anthocyanin values ranging from zero to 23 mg / 100 g fresh weight and from zero to 167.76 mg / 100 g dry weight [14, 15]. In fact, pigmentation is one of the main traits selected during the breeding of native Phureja landraces, producing an amazing diversity of coloration patterns, mostly associated with anthocyanin accumulation [16, 17].

**Fig 1:**
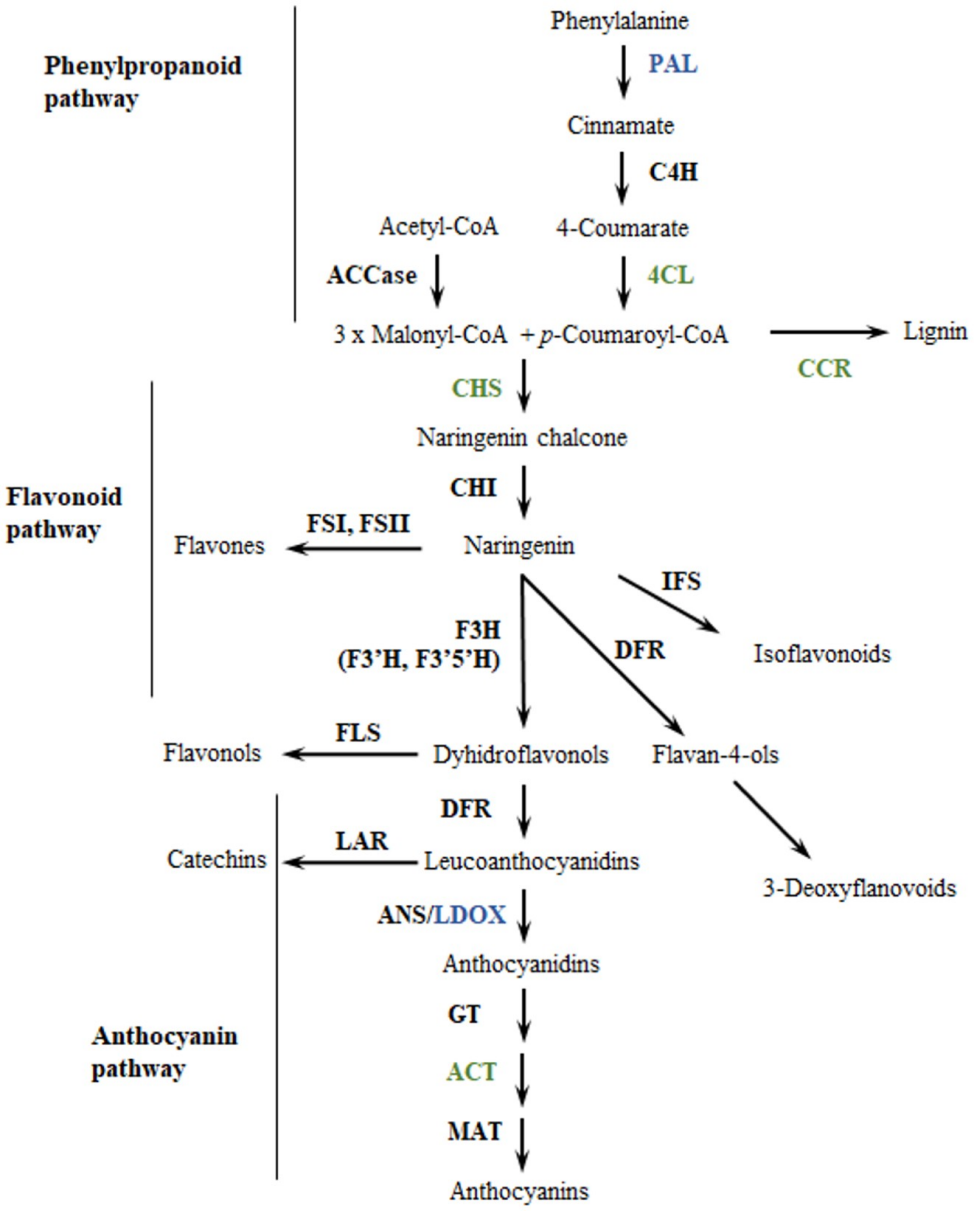
Enzymes from the phenylpropanoid pathway associated to anthocyanin variation in a population of *Solanum tuberosum* group Phureja. Enzymes in green correspond to genes identified in the gene-set analysis. Enzymes in blue correspond to genes identified in GWAS and gene-set analysis. Enzyme abbreviations: ACCase: acetyl-CoA carboxylase; PAL: phenylalanine ammonia-lyase; C4H: cinnamate 4-hydroxylase; 4CL: 4-coumarate:CoA ligase; CCR: cinnamoyl CoA reductase; CHS: chalcone synthase; CHI: chalcone isomerase; FS: flavone synthase; IFS: isoflavone synthase; F3H: flavanone 3β-hydroxylase; F3’H: flavonoid 3’-hydroxylase; F3’5’H: flavonoid 3’,5’-hydroxylase; FLS: flavonol synthase; DFR: dihydroflavonol 4-reductase; LAR: leucoanthocyanidin reductase; ANS: anthocyanidin synthase; GT: glucosyltransferase; ACT: anthocyanin acyltransferase; MAT: malonyltransferase. Modified from Springob [26].

Anthocyanins are synthesized in the cytosol through the phenylpropanoid pathway (Fig 1), which begins with the catalysis of the amino-acid phenylalanine by the enzyme phenylalanine-ammonia lyase (PAL). Then the chalcone synthase (CHS) catalyzes the condensation of three acetate units from malonyl-COA with p-coumaroyl-COA to yield tetrahydroxychalcone. Chalcone isomerase (CHI) then catalyzes the tetrahydroxychalcone to naringenin. Naringenin is hydrolyzed to dihydroflavonols by three enzymes, namely flavanone-3-hydroxylase (F3H), flavonoid-3´-hydroxylase (F3´H) and flavonoid-3´,5´-hydroxylase (F3’5’H). The dihydroflavonols are reduced to three different leucoanthocyanidins by dihydroflavonol-4-reductase (DFR), and their glycosylation by leucoanthocyanidin dioxygenase/anthocyanidin synthase (LDOX/ANS) produces the basic structures of anthocyanins (anthocyanidins—aglycons) that determine the coloration in plant tissues [18–21]. Genes encoding anthocyanin biosynthetic enzymes are known as “structural genes’’ and they are conserved among different species [22]. However, tissue-specific expression of different structural genes are controlled by transcription factors (TF) known as “regulatory genes’’ [22]. While structural genes determine the ability to produce a set of compounds regulatory genes generally affect the intensity and pattern of anthocyanin biosynthesis, particularly through the MYB-bHLH-WD (MBW) complex [23–25].

In potatoes the synthesis of anthocyanins was initially described as being controlled by the *R* (red), *P* (purple) and D/*I* (developer or inhibitor) loci, which map to chromosomes 2, 11, and 10, respectively [27–30]. *R* and *P* loci govern red and violet pigmentation in tubers while D/*I* is responsible for the intensity of pigmentation [27–30]. Later it was found that *R* and *P* loci code for two enzymes of flavonoid biosynthesis, DFR [31] and F3’5’H [32], which are responsible for the creation of red and purple anthocyanin pigments respectively. The locus *I* corresponded to a regulatory gene encoding the TF R2R3 MYB, which has a high similarity with the product of the *Petunia hybrida AN2* on chromosome 10 [33]. This gene governs the expression of multiple enzymes in the pathway, therefore affecting the level of multiple anthocyanin pigments [33]. Recently, a number of regulatory genes potentially controlling anthocyanin biosynthetic structural genes have been identified in potato tubers [20, 22, 34, 35] including three R2R3-MYB encoding genes (*StAN1*, *StMYBA1* and *StMYB113)* [20, 21, 36] two bHLH genes (*StJAF13* and *StbHLH1)* [21, 37] and one WD40 (*StWD40*) [38].

The pattern of anthocyanin composition across tissues and genotypes is controlled by multiple genes [18, 19]. Genome-wide association studies (GWAS) have been used to elucidate the complex genetic mechanisms that define anthocyanin content in potato tubers [15]. This methodology allows identifying quantitative trait locus (QTL) for a given trait and determining aspects of its genetic architecture, like the number of QTL and their respective contribution to the phenotype [27, 39, 40]. Pathway analysis can help exploiting the results of GWAS by using prior information on biological pathways and combining the genetic effects of many genes [41–43]. Different methods have been implemented to perform pathway-based analysis using data from GWAS [44]. Some studies use gene set enrichment analysis (GSEA) to examine whether a set of genes significantly associated with a trait of interest is enriched in specific pathways [45, 46]. Other analyses re-calculate associations in a predefined set of genes belonging to a biochemical route of interest [47]. A third approach analyses all genetic sequences associated with a trait of interest, regardless of significance or magnitude, and uses the gene effect values to calculate an enrichment score for each pathway [48–50]. Finally, one can combine analyses of multiple molecular or morphological traits evaluated in the same population to determine how these traits interact genetically [51].

Previous genetic studies of anthocyanin pigmentation in potato tubers conducted using biparental populations of potato identified a small number of QTL which explained from 8% to 11% of the phenotypic variation [27, 29, 51, 52]. Therefore, we still ignore the genetic factors that contribute to this missing heritability as well as the genomic distribution and biochemical identity of anthocyanin determinants. We also know little about the evolution of anthocyanin pigmentation during the domestication of potatoes. Has the same trait evolved repeatedly under different genetic control or does it have a unique origin across cultivated potatoes? The answers to these questions are crucial to design breeding strategies to improve anthocyanin content. Diploid landraces represent an underexploited source of genetic diversity [53–55] and an excellent model to fill these knowledge gaps. Recently, an exploratory analysis of anthocyanin content in these landraces identified QTL explaining more than 30% of the phenotypic variation [15]. Therefore, the primary objective of this study is to use pathway analysis to exploit previous studies conducted in the same population of diploid potatoes [15] to identify new genes, genomic regions and biochemical routes important for anthocyanin production. We were thus able to discover new genetic factors that drove for the recurrent evolution of anthocyanin pigmentation during the domestication of potato landraces. This information provides functional links to bridge the knowledge gap between the genetic variants and the phenotypes.

## Materials and methods

### Genome-wide association analysis

The Working Collection of Potato Breeding Program of *Solanum tuberosum* Group Phureja from the Universidad Nacional de Colombia (CCC) was employed for the GWAS using information partially published in previous studies [15]. Briefly, potato tubers from an association panel consisting of 96 accessions was phenotyped through Ultra High-Performance Liquid Chromatographic (UHPLC) analysis for the detection of five different anthocyanidins compounds (cyanidin, peonidin, pelargonidin, delphinidin, and petunidin) [15].

In order to do functional analyses, we expanded the SNP matrix used to run the GWAS in previous studies [15]. The original Single Nucleotide Polymorphism (SNP) matrix obtained through genotyping by sequencing [56] was filtered by removing SNPs with a minor allele frequency (MAF) higher or equal to 0.05 and less than 10% of missing data. We thus obtained 47,298 SNP markers.

The GWAS was conducted for each anthocyanin compound using a compression mixed linear model (CMLM) [57] applied by the Genome Association and Prediction Integrated Tool (GAPIT) R package ^®^ [58]. The principal components in the CMLM were used in order to control for population structure [59]. The Benjamini & Hochberg corrected threshold probability based on individual tests was calculated to control false-discovery rates (FDRs) [60] using a threshold of 0.1 given the sample size of the association panel (n = 96). The linkage disequilibrium (LD) between pairs of SNP markers was calculated through squared allele-frequency correlations (R^2^) by using TASSEL software [61].

### Gene-set analysis

We used *prior* biological information about the biosynthesis of anthocyanins to pre-select a subset of candidate genes. We searched in the literature for structural and regulatory genes involved in anthocyanin production in the Solanaceae family. We made use of a study that identified flavonoid orthologs in multiple Solanaceae species [62]. We also searched for genes associated with the term “anthocyanin” in the NCBI (https://www.ncbi.nlm.nih.gov/), KEGG (https://www.genome.jp/kegg/), Spud (http://solanaceae.plantbiology.msu.edu/), and BioCyc (https://biocyc.org/) genomic databases. In order to identify homologs of these anthocyanin genes in the potato genome we performed a BLASTx (v2.6.0) [63] of the sequences of from other plants against the potato reference genome DM—v4.03 [27, 64] and retrieved the best hits with a cutoff of 10^−20^. We then retrieved SNPs located ± 100 Kb of these genes and ran again the GWAS with this subset of SNPs. We used a relatively large window of 100 Kb in order to get multiple SNPs associated with the genes. The *p*-values within each gene were recalculated with the new subset of SNP markers and inputted to corrected for multiple testing using the approach reported by Benjamini and Hochberg [60], based on procedure to control FDR at 0.1. We assigned the lowest *p*-value value among all SNPs mapped to a gene as the p-value of the gene [65, 66]. The goal of this analysis was to identify anthocyanin homologs that show the strongest association with anthocyanin variation. For this reason, we evaluate only SNPs linked to anthocyanin homologs using standard GWAS methods. Therefore, significant SNPs are those that pass the significance and FDR thresholds using this reduced dataset.

### Pathway analysis

We used the PAST software [50] to conduct the pathway analysis using genomic annotation from two databases, PotatoCyc 4.0 (https://phytozome.jgi.doe.gov/pz/portal.html#!info?alias=Org_Stuberosum) and KEGG (https://www.genome.jp/kegg/pathway).

SNPs were assigned to genes based on LD information and a distance of 1,500 bp from the tagSNP [50, 67]. Statistical significance of a pathway was determined by taking 1,000 permutations of the gene effect values to generate a null distribution for the Enrichment Score (NES) [50]. Pathways with *p*-value < 0.05 were selected based on thresholds set for gene association and effect values of the genes.

### Phylogenetic and population genetics analyses

We used the TASSEL software to conduct a phylogenetic tree of the populations using the Neighbor Joining algorithm and two inputs: (1) All SNPs used in GWAS and (2) significant SNPs from Chromosome 10. We also used Tassel to conduct principal components analysis (PCA) using the covariance option and the same sets of SNPs.

TASSEL was used to calculate the Tajima D statistic using the sliding window option (step = 10, window = 10). SNPs falling in the upper and lower 1% percentiles of the distribution were considered candidates for balancing and positive selection respectively (S11 Table).

### Genomic architecture of variation for other traits

The potato population used in our study has also been rated for other agricultural and nutritional important traits like macronutrients [68], sugars [54], hydroxycinnamic acids (HCAs) [69], and resistance to late blight caused by the pathogen *Phytophtora infestans* [56]. We were interested in evaluating whether some of these traits show phenotypic or genomic correlations with anthocyanin variation. We ran a PCA using phenotypic data and calculated pairwise correlations between all traits using TASSEL. We used TASSEL to conduct GWAS for all traits using a mixed linear model (MLM), correcting for population structure with a PCA (covariance method, 5 components) and a Centered IBS method of Kinship estimation. Each variance component was estimated once (P3D).

## Results and discussion

### Genome-wide association in an extended SNP panel identifies new candidate genes

In this study we exploited previous research on the Work Collection of Potato Breeding Program from Colombia [15] to understand the genomics and evolution of anthocyanin variation. The population analyzed here is relatively small but genetically diverse [53, 70], thus representing a valuable resource to identify and manipulate traits that have been lost during the breeding of cultivars outside the original range of potatoes [4, 5]. We re-analyzed data from a GWAS of five anthocyanin compounds, namely cyanidin, peonidin, pelargonidin, delphinidin, and petunidin [15], using an expanded dataset of 47,298 SNP markers (S1 Table). LD decay is fast in this population (S3 Table) which makes it a good system to track causal genes. Therefore, by expanding the genotyping panel we were able to search for genes underlying the QTL detected previously and find new important variants. In total 22 SNPs were significantly associated with at least one compound at a genome-wide FDR of 0.1 (Table 1, S1 Fig and S2 Table). Sixteen of these significant SNPs were located in the coding region of annotated genes on the Chromosomes 1, 2, 6, 9, 10 and 11 (Table 1).

**Table 1:** Summary of genome associations for anthocyanin content in a population of *Solanum tuberosum* group Phureja.

Trait	Chr	Position	R^2^ model	*p*-value	FDR_Adjusted	Effect	Gene_ID	Annotation gene
All anthocyanins	ch10	52004868	0,44	6.21E-09	2.9,E-04	0.0406	PGSC0003DMG400017604	Chloroplast threonine deaminase
Pelargonidin, peonidin, delphinidin	ch10	52261553	0,45	8.35E-08	0.002	-0.4556	PGSC0003DMG400017597	STS14 protein
Pelargonidin, delphinidin, peonidin	ch10	52261573	0,46	4.54E-08	0.002	-0.0506	PGSC0003DMG400017597	STS14 protein
Delphinidin, peonidin, pelargonidin	ch10	52004940	0,40	1.42E-07	0.0029	-0.364	PGSC0003DMG400017604	Chloroplast threonine deaminase
Peonidin, delphinidin, pelargonidin	ch10	54746624	0,35	2.07E-06	0.0196	-0.1967	PGSC0003DMG400011047	60S ribosomal protein L4/L1
Petunidin	ch06	8156658	0,19	2.77E-06	0.0262	0.4535	PGSC0003DMG400025399	Vacuolar membrane protein PEP3
Petunidin	ch06	8156696	0,19	2.77E-06	0.0262	-0.1985		
Petunidin	ch01	72364545	0,18	3.34E-06	0.0263	-0.2882	PGSC0003DMG402000051	HMG-I and HMG-Y
Pelargonidin	ch02	41058521	0,37	5.06E-06	0.0342	0.0631	PGSC0003DMG400012655	Nadph-cytochrome P450 oxydoreductase
Pelargonidin	ch02	41058534	0,37	5.06E-06	0.0342	-0.4182	PGSC0003DMG400012655	DUF292 domain containing protein
Pelargonidin	ch02	41058575	0,37	5.06E-06	0.0342	0.141	PGSC0003DMG400012655	DUF292 domain containing protein
Petunidin	ch01	24135132	0,19	7.37E-06	0.0498	0.0975		
Petunidin	ch01	24135105	0,19	9.94E-05	0.0588	0.0052		
Peonidin, delphinidin	ch10	54778485	0,33	7.67E-06	0.0605	0.1559	PGSC0003DMG400010985	Subtilase
Petunidin	ch11	3129502	0,18	1.29E-05	0.0679	-0.0546		
Pelargonidin	ch10	51318001	0,35	1.40E-05	0.0829	0.0855	PGSC0003DMG400019155	F-box family protein
Petunidin	ch01	53094258	0,19	2.28E-05	0.0982	0.1819	PGSC0003DMG400023276	60S ribosomal protein L27
Petunidin	ch01	53094259	0,19	2.28E-05	0.0982	0.3271	PGSC0003DMG400023276	60S ribosomal protein L27
Cyanidin	ch01	62416352	0,19	2.55E-05	0.0996	0.068	PGSC0003DMG400009033	Myb 12 transcription factor
Petunidin	ch11	33265607	0,19	1.31E-04	0.0996	-0.3339	PGSC0003DMG400008650	Leucoanthocyanidin dioxygenase LDOX
Cyanidin	ch09	59749919	0,19	2.54E-05	0.1008	-0.0838	PGSC0003DMG400020593	Acyl-CoA synthetase
Pelargonidin	ch10	54207412	0,19	3.29E-05	0.1011	0.0527	PGSC0003DMG400011082	PRA1 family protein

Our results confirmed the significant SNPs reported by Parra-Galindo [15] and identified new associations. The strongest association signals were detected again in two nearby defensive genes from Chromosome 10, namely within the *Chloroplast threonine deaminase 1* (*PGSC0003DMG400017604*) and the *STS14* (*PGSC0003DMG400017597*) genes. Chloroplast threonine deaminases are the first enzymes in the biosynthesis of the amino acid isoleucine but also mediate plant defenses against pathogens and herbivores [71]. STS14 belongs to a family of pathogenesis-related secretory proteins [72] Although anthocyanin compounds are induced by biotic stress these two enzymes have not been associated to anthocyanin production previously. It is thus possible that causal mutations underlying these associations are positioned in other genes located in the vicinity, as we will explore in the next sections.

We highlight three newly detected associations from our expanded SNP set involving polymorphisms located within putative anthocyanin biosynthetic genes (Table 1); a SNP on Chromosome 11 linked to the *Leucoanthocyanidin dioxygenase* gene *(LDOX; PGSC0003DMG400008650)*; a position on Chromosome 1 located in a R2R3-MYB transcription factor *(Myb12; PGSC0003DMG400009033)*; and a SNP on chromosome 6 linked to the *Vacuolar membrane protein* (*PEP3; PGSC0003DMG400025399*). Firstly, *LDOX* enzyme, also known as anthocyanidin synthase, catalyzes the conversion of leucoanthocyanidins to anthocyanidins, the precursors of anthocyanins [73]. Previous studies [74, 75] found that *LDOX* genes are located on QTL for anthocyanin variation in Chromosomes 8 and 9. However, there are no reports of anthocyanin QTL in the genomic region of Chromosome 11 containing the *LDOX* gene reported in our study. Secondly, R2-R3 MYB transcription factors showing homology with *Petunia AN2* gene (Borevitz2000) regulate anthocyanin biosynthesis in dicotyledonous plants [76–80]. A number of these AN2 homologs regulate the expression of anthocyanin enzymes in tubers and flowers of potato [33, 35, 37], including three nearby genes from chromosome 10 named *StAN1*, *StAN2 and StFlAN2*. The Myb12 TF detected in this study has not been linked to anthocyanin production in potatoes but it´s orthologs regulate anthocyanin production in other plants like *Arabidopsis thaliana* [81], apple [82], lily [83, 84], and grape [85]. Finally, the *Vacuolar membrane protein PEP3* is an interesting candidate because this gene is involved in vacuole organization [86], a process that is essential for anthocyanin biogenesis and accumulation.

By expanding the number of genetic markers evaluated in this potato population and analyzing the function of genes underneath the most associated markers from GWAS we were able to identify potential determinants of anthocyanin variation in potatoes that were not detected in previous studies. Given that causal genes might be in the vicinity of significant SNPs we searched for anthocyanin homologs in broader QTL regions.

### Analysis of anthocyanin homologs provides a deeper understanding of pathway regulation

The anthocyanin biosynthetic pathway is one of the most extensively studied pathways of plant specialized metabolism. Several genes have been reported to determine anthocyanin levels in potato cultivars and accessions [20, 21]. However, we know little about the causes of anthocyanin variation in potato landraces, which have a broader and largely untapped genetic diversity [5]. We made use of the extensive information available in the literature and genomic databases on anthocyanin genes in other plants to identify gene targets contributing to anthocyanin variation in this genetically diverse panel of potato landraces. In GWAS, it is a challenge to identify variants with moderate to weak effect sizes because the effect of many variants can be compounded by interactions with other loci [39]. In order to identify genes contributing to the missing heritability in anthocyanin accumulation we re-evaluated trait associations using only SNPs genetically linked to a set of anthocyanin homologs. Specifically, we tested for an association between the content of five anthocyanins in a subset of pre-selected candidate anthocyanin homologs retrieved from the literature and databases (S4 Table). The analysis involved recalculating trait associations among the five anthocyanins and the genotypes at SNP markers located within ± 100 Kb of the 108 *anthocyanin homologs* (S5 Table). Nineteen SNPs were statistically significant at 0.1 FDR for at least one of five anthocyanin compounds (S6 Table). These 19 significant SNP markers were located into 10 of the pre-selected candidate genes (Table 2). Nine of these SNPs were not detected in the initial genome-wide association study.

**Table 2:** 

RefGen_DM -V4.03	Annotation gene	Compound	Chr	Position	*p*-value	FDR_Adjusted *p*-value
PGSC0003DMG400031365	Phenylalanine ammonia-lyase	All anthocyanins	Ch10	52,004,868	8,14 x 10^−9^	0,000555
PGSC0003DMG400019825	Cinnamoyl-coA reductase	Pelargonidin	Ch03	56,864,063	2,17 x 10^−6^	0,002551
PGSC0003DMG400034671	1-O-acylglucose:anthocyanin-O-acyltransferase	Cyanidin	Ch04	68,747,095	2,17, x 10^−6^	0,002551
PGSC0003DMG400010987	Wrky transcription factor	All anthocyanins	Ch10	54,746,624	2,09 x 10^−6^	0,002599
PGSC0003DMG400024129	Leucoanthocyanidin dioxygenase	Petunidin	Ch01	15,307,188	4,22 x 10^−5^	0,013962
PGSC0003DMG400003155	4-coumarate:CoA ligase	Petunidin	Ch03	47,051,715	2,15, x 10^−4^	0,028549
PGSC0003DMG400029620	Chalcone synthase	Petunidin	Ch09	58,297,746	2,15 x 10^−4^	0,043449
PGSC0003DMG400025373	Cinnamoyl-coA reductase	Cyanidin	Ch11	44,394,705	9,24 x 10^−4^	0,058597
PGSC0003DMG400006814	AN1-like transcription factor	Cyanidin	Ch01	66,008,979	1,48 x 10^−3^	0,098137
PGSC0003DMG401012339	BHLH transcription factor JAF13	Cyanidin	Ch08	54,831,215	1,65 x 10^−3^	0,099547

Seven structural genes showed significant associations in this analysis (Table 2). In Fig 1 we show their positions within the Flavonoid pathway. These were: *phenylalanine ammonia-lyase (PAL)*; leucoanthocyanidin dioxygenase *(LDOX*); cinnamoyl-coA reductases *(CCR1*); 4-coumarate: CoA ligase (*4CL1*); 1-O-acylglucose:anthocyanin-O-acyltransferase enzyme *(CtSCPLAT1*); and chalcone synthase enzyme *(CHS*) [18]. CCR is considered a control point in regulating the overall carbon flux toward lignin [87] and downregulation of CCR activates the enzymes PAL, C4H and 4CL [88]. The 4-coumarate-CoA ligase is a key enzyme in the phenylpropanoid pathway participating in monolignol biosynthesis [89] while the CHS catalyzes the first committed step in the biosynthesis of anthocyanin pigments [18]. *CHS* enzymes located within anthocyanin QTL have been identified in tomato (*TCHS1*, *TCHS2*) [90] and potato [74]. Finally, the 1-O-acylglucose:anthocyanin-O-acyltransferase enzyme catalyzes the acylation reactions of anthocyanins one of the final steps in anthocyanin biosynthesis [91].

A *phenylalanine ammonia-lyase* homolog (*PGSC0003DMG400031365*) in chromosome 10 is particularly interesting because it is linked to the most significant SNPs that display associations to the five anthocyanins measured. The association of a PAL homolog to all anthocyanins makes sense biochemically because the PAL enzyme catalyzes the first reaction in the phenylpropanoid biosynthetic pathway [92], which leads to the biosynthesis of all anthocyanins (Fig 1). The role of PAL homologs in the regulation of anthocyanin production has been described in many plants [93–95]. For instance, in *A*. *thaliana*, mutations in the two isoforms of *PAL* gene cause a reduced production of all anthocyanins [96]. Within the potato genome there are 11 *PAL* genes but only the homolog identified in our study has been previously associated with variation in anthocyanin content. For instance, Liu and colleagues [97] found that changes in the expression of this gene are involved in anthocyanin biosynthesis under heat stress. Additionally, the upregulation of this gene is associated with a greater accumulation of anthocyanins in potato flowers [35].

Importantly, eight significant SNP markers were located near the *PAL* gene, in a region of 4 Mb in the extreme of Chromosome 10 (Fig 3). These SNPs were linked to anthocyanin regulatory genes, namely *StFlAN2* (*PGSC0003DMG400019217*) and *WRKY 13* (*PGSC0003DMG400010987*) (Table 2). It was recently discovered that StFlAN2 is the main regulator of floral anthocyanin production in potato [35], matching the function of its ortholog in petunia (*PhAN2*). On the other hand, *WRKY 13* is orthologous with *TRANSPARENT TESTA GLABRA 2* from Arabidopsis and PhPH3 from petunia, which control the transcription of structural genes responsible for anthocyanin biosynthesis as well as ion pumps that determine the pH of vacuoles, where anthocyanins are stored [98]. Interestingly, the transcription factors identified in our study are members of three families known to control anthocyanin production through the formation of the so-called MBW complex [25]: a MYB TF (*StFlAN2*), a BHLH TF (*JAF13*) and a WRKY TF (*WRKY 13*). Furthermore, the orthologs of these genes interact to control anthocyanin production and storage in other plant species [36]. These results show that, by integrating previous information on pathways we were able to recover a more complete picture of the gene interactions that determine anthocyanin variation in potatoes.

#### Anthocyanin genes are clustered in Chromosome 10

Many of the QTL identified in studies of anthocyanin variation simultaneously govern variation in multiple anthocyanin compounds [33, 76, 99]. The co-localization of QTL often arises from the existence of pleiotropic genes governing the biosynthesis of multiple pigments [99] but can also result from genetic clustering of determinants of the different anthocyanins [100]. In this collection of potatoes, the levels of the five different anthocyanins are correlated across individuals [15]. Additionally, the most significant SNPs from GWAS govern variation in multiple anthocyanins and are located in a relatively small (4 Mb) region at the end of Chromosome 10 and (Tables 1 and 2, Fig 3). These results can be explained by the existence of pleiotropic genes and/or by the presence of anthocyanin biosynthetic clusters in Chromosome 10. In this context, we define a pleiotropic gene as a gene that governs simultaneously the production of multiple anthocyanins while a biosynthetic cluster is a physically clustered group of two or more genes that together determine the production of anthocyanins. We evaluated these two non-exclusive hypotheses by analyzing gene function, recombination and genetic variation in this 4 Mb genomic region.

We first looked at the distribution of anthocyanin homologs across chromosome 10 to see if these putative anthocyanin genes are clustered in the 4 MB region containing significant SNPs from GWAS. We found that this genomic region contains 10 out of the 28 putative anthocyanin genes located in Chromosome 10 and is among the regions of the genome with the highest density of anthocyanin homologs (Fig 3, S10 Table). These include the *PAL* gene, four putative 7-O-linked N-acetylglucosamine transferases, an oxidoreductase, a WRKY transcription factor, and at least three Myb transcription factors (S6 Table). Some of these genes are adjacent and seem to be the result of recent tandem duplications. These include PAL [97], 7-O-linked N-acetylglucosamine transferases and Myb transcription factors. In fact, previous studies have shown that this genomic region is very dynamic, with both transposon activity and copy number variation [35]. This genomic region contains the MYB TF StFlAN2 regulating flower color as well as a close paralog also responsible for segregation of corolla anthocyanin production. More importantly, the genomic region also contains two additional R2R3 MYB TFs that determine anthocyanin production in potato tubers (StAN1) [33] and throughout the plant (StAN2) [36].

We calculated LD in the 4 Mb genomic region, as high LD can indicate the maintenance of a cluster of linked alleles that are inherited as a single haplotype [101]. LD decay is relatively fast in this genomic region (mean distance among markers with a R^2^ > 0.8 for this genomic region = 11,935 ± 5,007, for the rest of the chromosome = 21,130 ± 1,217). This indicates that recombination is not reduced at this site and that different significant SNPs from GWAs are located within different haplotypes (mean distance between SNPs = 7,422 ± 1,965) (S7 Table).

**Fig 2:**
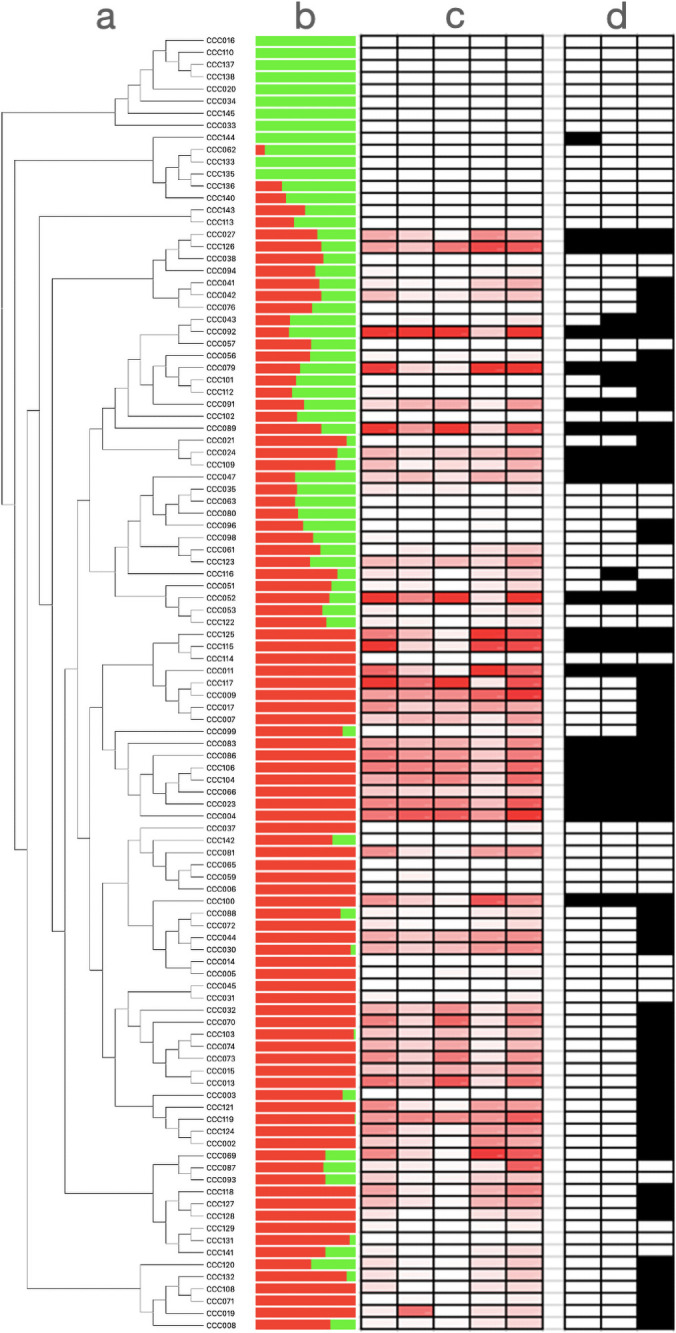
Genetic and metabolic variation shown in *Solanum tuberosum* group Phureja. (a) Neighbor joining phylogeny conducted with all SNPs used in GWAS. Names on the top of the branches correspond to the names of potato accessions from the Colombian Core Collection (CCC). (b) Results of Admixture with a K = 2. (c) Anthocyanin content: Darker colorations are associated with darker concentrations of the compound. 1: Delphinidin; 2: Cyanidin; 3: Petunidin; 4: Pelargonidin; 5: Peonidin. (d) Genotypes at significant SNPs from Chromosome 10. The allele associated to a darker coloration (in homozygous or heterozygous state) is in black. 1: pos 52,004,868; 2: pos 52,261,553; 3: pos 54,746,624.

**Fig 3:**
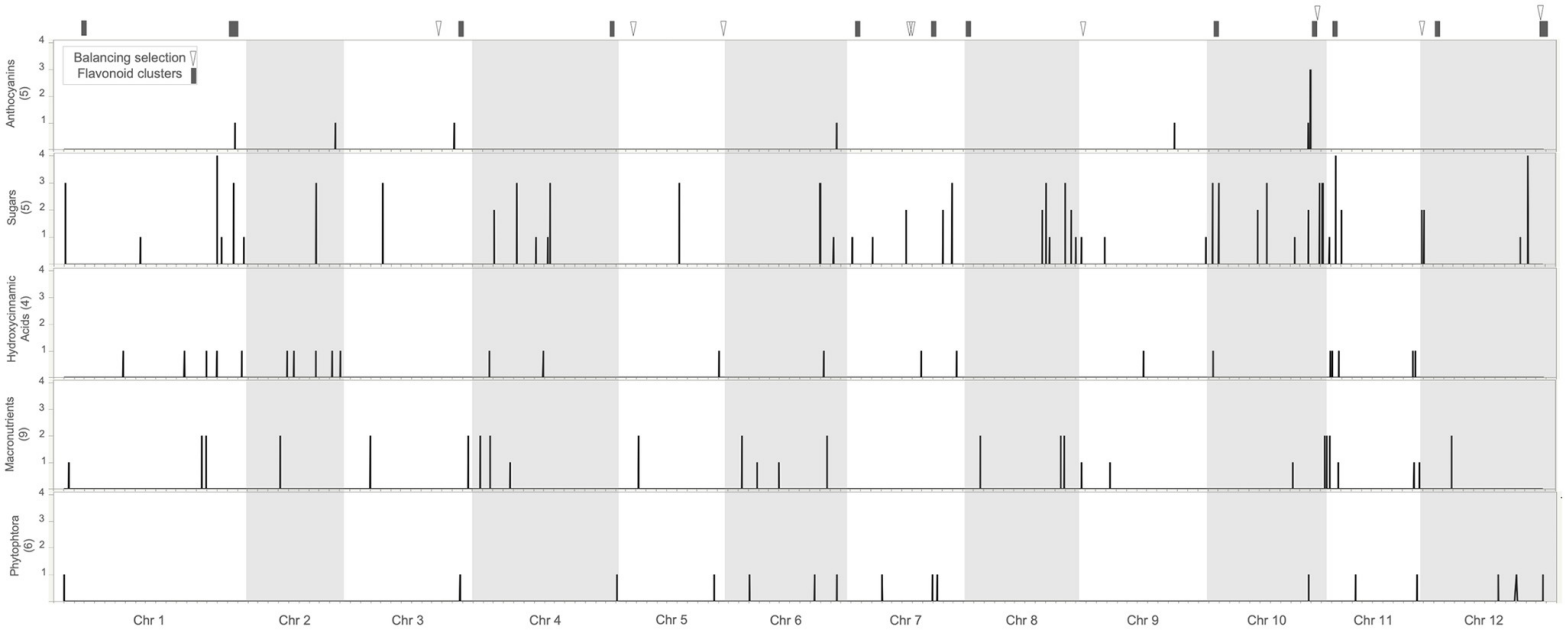
Genomic architecture of variation in anthocyanins and other agronomic traits. Each horizontal panel corresponds to a set of traits, with the number of traits in the set indicated in parenthesis. In the X axis we present the position of the SNPs across the genome and in the Y axis we show the number of traits significant for a particular SNP. In the top we indicate the location of 1 Mb intervals showing clusters (>3 genes) of flavonoid genes as well as footprints of balancing selection according to Tajima’s D statistic.

It is likely that the genetic basis of anthocyanin variation differs across individuals from our panel. To evaluate whether potatoes with high anthocyanin content shared alleles at the loci governing anthocyanin variation, we analyzed the genomic variation of significant SNPs from GWAS using phylogenetic and multivariate analyses. We found that the genotypes at significant SNPs from Chromosome 10 do not fully separate plants with high and low anthocyanin content (Fig 2, S2 Fig). This suggests that anthocyanin variation has multiple phylogenetic and genetic origins in the population. Finally, we searched for footprints of natural selection in this genomic region using Tajima’s D statistic. Values of Tajima’s D are high (i.e., upper 99 the percentile of genome wide distribution) at the edge of the putative anthocyanin cluster (Fig 3, S1 Table). This indicates that alleles are kept at intermediate frequencies, a genomic pattern that can result from balancing selection.

Pleiotropy and genetic clustering are common genomic patterns in specialized metabolism [102–105]. Both patterns can produce correlations between the concentrations of different metabolites. Pleiotropy usually involves enzymes acting upstream in the biosynthetic pathway or regulatory genes governing the expression of key enzymes [102, 106, 107]. We found evidence of pleiotropy since the most significant SNPs from GWAS are associated to the content of all anthocyanins. Additionally, these SNPs are linked to putatively pleiotropic genes. For instance, the *PAL* gene catalyzes the first step in the phenylpropanoid pathway and therefore could be a pleiotropic gene whose expression or/and sequence affects the production of anthocyanins located downstream in the biosynthetic pathway. Intriguingly, *PAL* is located nearby three pleiotropic transcription factors that govern the production of multiple anthocyanins *StAN1*, *StAN2 StflAN2*. Another candidate from Chromosome 10 that could pleiotropically control multiple anthocyanins is the *WRKY* 13 TF [25]. It should be mentioned that given the fast LD decay of the population it is not likely that the GWAS signal comes from any of these genes exclusively.

The clustering of genes from the same route has been reported in many specialized metabolism pathways and it is proposed as a mechanism to synchronize gene expression or to maintain favorable allelic combinations in the face of recombination [103, 104]. Despite the tandem duplication of many enzymes and TFs involved in anthocyanin metabolism some studies suggest that this pathway is particularly reticent to clustering [108, 109]. Surprisingly, we found evidence of clustering of anthocyanin determinants, as there is a concentration of structural and regulatory genes in the region of Chromosome 10 containing significant SNPs from GWAS. Many of these genes are located at very short distances from each other and present polymorphic tandem duplications and deletions in the potato lineage [64, 110], including the *PAL* gene [97] as well as the 7-O-GTs [62] and MYB transcription factors [37, 64, 110]. Finally, the genes from this putative cluster show coordinated expression patterns [97], which suggest that they are under a common genetic control. This genomic region is remarkably dynamic in potatoes as well as in other Solanaceae like petunia [111] and tomato [112], perhaps due to high transposon density. This suggests that natural selection as well as domestication have shaped this region concentrating anthocyanin determinants.

Phylogenetic analyses of the genomic region containing this group of anthocyanin genes suggest that multiple alleles or allelic combinations were involved in the creation of potato landraces with high anthocyanin content. Interestingly, a study of historical European samples shows a drastic reduction of genetic diversity and negative values of Tajima´s D in this genomic region [113]. The authors associate this pattern to selection in gibberellin genes during the adaptation of potatoes to European temperate weathers after their introduction from South America [113]. This is in contrast with Elevated Tajima´s D found in our study, which indicates that South American varieties maintained high genetic diversity in this genomic region. We postulate that this genetic diversity could have resulted from selection for diverse patterns of tuber coloration during the domestication and improvement of potato landraces in the Andes.

### Amino acid metabolism and sugar metabolism are associated to anthocyanin variation according to pathway analysis

Anthocyanin variation is a complex trait determined by interactions among genes that influence the expression of each specific compound. Pathway analyses can help identifying genes with small effects in the phenotype by using previous functional annotation to identify pathways that are enriched in genes with high GWAS associations [114]. We used the PAST software to conduct pathway analyses [50] by using gene annotation from KEGG and PotatoCyc databases. SNPs used in GWAS were assigned to 8,833 genes based on LD information. The genes were associated with 111 PotatoCyc pathways, and 104 sot-KEGG pathways. We thus identified 22 significantly enriched pathways in the GWAS of anthocyanin content in potato tubers *(p*-value < 0.05, Table 3, S8 Table).

**Table 3:** Summary of the pathway-based analysis for pathways with *p*-value < 0.03 to five anthocyanin compounds in *Solanum tuberosum* group Phureja.

Trait	Data Base	ID	PW Name	*p*-value	NES	Genes^a^
Pelargonidin	Potatocyc	PWY-6441	Spermine and spermidine degradation III	0,0117	0,67	5
Pelargonidin	Potatocyc	PWY-6596	Adenosine nucleotides degradation I	0,0143	0,71	9
Pelargonidin	KEGG-sot	sot00260	Glycine, serine and threonine metabolism	0,0144	0,40	24
Pelargonidin	KEGG-sot	sot01230	Biosynthesis of amino acids	0,0177	0,27	67
Cyanidin	Potatocyc	PWY-2261	ascorbate glutathione cycle	0,0078	0,67	7
Cyanidin	Potatocyc	PWY-702	L-methionine biosynthesis II	0,0111	0,6	8
Cyanidin	Potatocyc	LEUSYN-PWY	L-leucine biosynthesis	0,0212	0,67	11
Cyanidin	Potatocyc	PWY-6441	Spermine and spermidine degradation III	0,0201	0,63	9
Cyanidin	KEGG-sot	sot00030	Pentose phosphate pathway	0,0105	0,58	10
Cyanidin	KEGG-sot	sot00520	Amino sugar and nucleotide sugar metabolism	0,0111	0,41	24
Cyanidin	KEGG-sot	sot00900	Terpenoid backbone biosynthesis	0,0235	0,41	20
Peonidin	Potatocyc	PWY-6441	Spermine and spermidine degradation III	0,0201	0,62	9
Peonidin	KEGG-sot	sot00030	Pentose phosphate pathway	0,0227	0,56	10
Delphindin	Potatocyc	PWY-6441	spermine and spermidine degradation III	0,0041	0,77	8
Delphindin	Potatocyc	PWY-702	L-methionine biosynthesis II	0,0211	0,59	8
Delphindin	KEGG-sot	sot00030	Pentose phosphate pathway	0,0206	0,58	9
Petunidin	Potatocyc	PWY-7184	Pyrimidine deoxyribonucleotides biosynthesis I	0,0088	0,75	8
Petunidin	Potatocyc	PWY-702	L-methionine biosynthesis II	0,0159	0,61	8
Petunidin	Potatocyc	GLUCOSE	Glucose and glucose-1-phosphate degradation	0,0250	0,63	6
Petunidin	KEGG-sot	sot00030	Pentose phosphate pathway	0,0233	0,53	10

PW pathway, NES normalized enrichment score.

^a^The number of genes that were mapped to a pathway and contributed to the enrichment score calculation.

We found an enrichment of genes involved in biosynthesis of methionine and sugars and the degradation of spermidine and spermine. An association between the biosynthesis of the amino acid methionine and anthocyanin has been previously reported in many plants [115–117]. For instance, Dancs and colleagues [118] found that over-expressing a gene involved in methionine synthesis induced a decrease of the expression of *PAL*, which caused a reduction in the amounts of anthocyanin pigments in mutant potato tubers. On the other hand, the catabolism of the sugar glucose via the pentose phosphate pathway (PPP) also has been associated with anthocyanin production in fruits [119]. Stimulating PPP activity in fruits induces an increase in anthocyanin content since some products of the PPP are essential precursors for the production of anthocyanins [120, 121]. The enzyme Glucose-6-phosphate dehydrogenase (G6PDH) plays a particularly important role in this crosstalk between the primary and secondary metabolism and shows a correlation with the levels of mRNAs encoding PAL and CHS [122]. Finally, the hormone ethylene, which regulates anthocyanin biosynthesis during senescence and stress [122], is derived from spermidine and spermine [123, 124]. Pathway analysis thus revealed plausible links between anthocyanin production and other metabolic and signaling pathways. Interestingly, according to the literature the PAL enzyme is important to establish these physiological tradeoffs, which could explain its strong association to anthocyanin variation in our study. Given that tradeoffs between the primary and secondary metabolism can impact agronomical attributes we evaluated if anthocyanin content is genetically correlated to other important traits in our potato collection.

### Anthocyanin content is correlated to other agronomic traits

We analyzed phenotypic variation for multiple agronomically important traits to identify pathways associated with anthocyanin production. We first evaluated pairwise correlations (S9 Table) between the levels of anthocyanins, macronutrients, sugars, hydroxycinnamic acids (HCAs), and resistance to late blight. We found that the levels of all anthocyanins are positively correlated, which is consistent with the co-localization of significant SNPs for the different compounds and with the linkage between these SNPs and the *PAL* gene. We also found significant correlations between the levels of anthocyanins with tuber content of HCAs (chlorogenic acid, crypto-chlorogenic acid and neo-chlorogenic acid). This correlation is not surprising given that HCAs are chemically conjugated to anthocyanins and anthocyanin-linked HCAs are frequently reported in red skin or flesh of potato tubers [125, 126].

We also analyzed the genomic location of significant SNPs identified with GWAS for the different traits (Fig 3). We found that the putative anthocyanin cluster at chromosome 10 also contains SNPs associated with resistance to *P*. *infestans* and sugars content. We also identified positions in chromosomes 1, 2, and 6 governing simultaneously anthocyanins and other traits (Fig 3). The co-localization of QTL for anthocyanins and sugars supports our results of pathway analysis linking the PPP to anthocyanin variation and is consistent with the biochemistry of anthocyanins, which are sugar-decorated [127]. The colocalization of QTL for anthocyanin variation and resistance to late blight is consistent with recent studies showing that anthocyanins have played a role in mounting defenses against Oomycete infection since the divergence of land plant lineages [128]. Overall, these results suggest that breeding strategies aimed at increasing anthocyanin content will likely cause changes in other important traits. This highlights the importance of maintaining genetic diversity to evaluate combinations of genetic variants that produce the most favorable phenotype.

## Conclusions

In natural populations, genome wide association studies allow to explore the genetic architecture of complex traits. Here we used accessions of diploid potatoes to identify structural and regulatory genes associated with five anthocyanins. Among these genes, we highlight a *PAL* gene on Chromosome 10 associated with the five-anthocyanin compounds. This gene is contained in a region on chromosome 10 that also harbors other significant SNPs as well as multiple anthocyanin homologs. These results highlight the value of using a diverse collection of native landraces: On one hand genes like *PAL* which are pleiotropic and show evidence of recurrent selection are excellent targets for breeding programs because they have repeatedly tested by selection and produce big changes in the phenotype. The short distance between this gene and multiple MYB TFs associated with anthocyanin regulation in potato, proves that loci identified in QTL mapping can contain multiple causal genes. On the other hand, varieties that do not contain selected variants at these loci can be used to identify novel anthocyanin determinants that could help improve the concentration or expression patterns of anthocyanins during tuber development.

Given that potatoes with high anthocyanin content have multiple origins, we wanted to evaluate if selection in the same alleles or haplotypes at this cluster was involved in the repeated breeding of potatoes with high anthocyanin content. We found that most potatoes with high anthocyanin content share the same genotypes at this cluster, suggesting that there was recurrent selection on the same alleles. However, according to phylogenetic analyses the accumulation of anthocyanin seems to also have involved other alleles. Accordingly, this region has high diversity, consistent with balancing artificial selection to breed varieties with diverse colors.

Finally, we integrated data from multiple traits and used a pathway analysis to find candidate pathways that might be underlying anthocyanin accumulation in potato tubers. The results of this analysis revealed a putative relation between anthocyanin regulation in diploid potato and the biosynthesis of methionine, sugars and hydroxycinnamic acids. The knowledge gained with this complementary analysis has improved the understanding of differences in anthocyanin accumulation and can help identify strategies for increasing anthocyanin production through physiological manipulation, genomic selection, or metabolic engineering.

## Supporting information

S1 Fig Genome wide association study of five anthocyanin compounds in diploid potato. Manhattan Plots of association results from a compression mixed linear model of each anthocyanidin. Negative log10-transformed P-values (y-axis) from a GWAS are plotted against physical position (DM_v4.04_pseudomolecules) on each of 12 chromosomes.
(TIF)

S2 Fig Multivariate analysis of genetic variation in the association panel. Principal components analysis of all SNPs used in the GWAS. The color of each dot indicates the total anthocyanin content (arithmetic sum of the five anthocyanins) for each plant.
(PDF)

S1 Table Data set of 47,298 SNP markers array. For each polymorphic site we present the diploid genotype of the 96 accessions used.
(XLSX)

S2 Table Results from the genome-wide association study of 5 anthocyanin compounds in diploid potatoes. Analyses were run with GAPIT using an array of 47,298 SNP markers.
(TXT)

S3 Table Linkage disequilibrium. Calculated with Tassel for the 47,298 SNP markers evaluated in 96 potato genotypes.
(TXT)

S4 Table Genomic information for the subset of 108 candidate genes. Putative anthocyanin genes retrieved from the literature.
(XLSX)

S5 Table GWAS results obtained for SNP markers located nearby anthocyanin genes. GWAS was run using only SNPs mapped within ± 100 Kb of 108 anthocyanin homologs.
(XLSX)

S6 Table Statistically significant results of gene-set analysis. Genome-wide association of 5 anthocyanin compounds in SNPs located nearby a pre-selected set of 108 candidate genes.
(XLSX)

S7 Table LD data. For each marker we calculated the maximum distance in bp to markers associated with a minimum correlation (r2) of 0.8.
(XLSX)

S8 Table Association effect and p-values for tagSNPs and genes in the pathways with p-value < 0.05. Results from the PAST software using GWAS results and gene annotation from the PotatoCyc and KEGG databases.
(XLSX)

S9 Table Trait correlations in mapping populations. Pairwise correlation between traits previously evaluated in this population, namely anthocyanin, macronutrients, sugars, Hydroxycinnamic acids (HCAs), and resistance to late blight.
(TXT)

S10 Table Distribution of anthocyanin genes in the potato genome. Number and density of anthocyanin homologs across non-overlapping 1 Mb intervals.
(TXT)

S11 Table Tajima’s D across the diploid potato genome. TASSEL was used to calculate Tajima’s D statistic as well as genetic diversity (pi and theta) using a sliding window (step = 10, window = 10). SNPs falling in the upper and lower 1% percentiles of the distribution were considered candidates for balancing and positive selection respectively.
(TXT)
