# Supplementary figures and images for: Pathway-based analysis of anthocyanin diversity in diploid potato
Source: PLoS One. 2021 Apr 29;16(4):e0250861. doi: 10.1371/journal.pone.0250861 (PMC8084248; doi:10.1371/journal.pone.0250861)

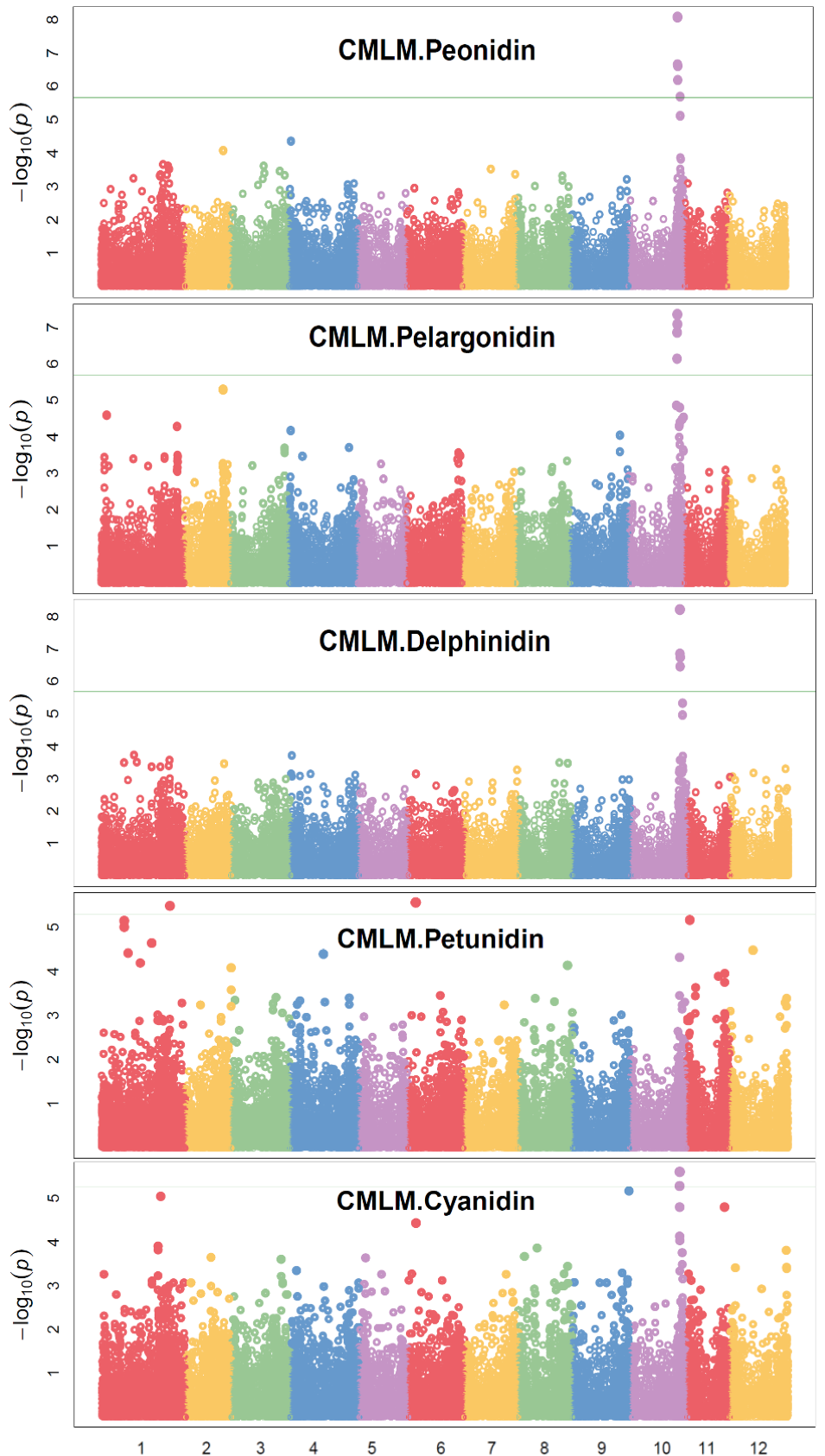

Supplement: S1 Fig — Manhattan Plots of association results from a compression mixed linear model of each anthocyanidin. Negative log10-transformed P-values (y-axis) from a GWAS are plotted against physical position (DM_v4.04_pseudomolecules) on each of 12 chromosomes. (TIF) [file pone.0250861.s001.tif]

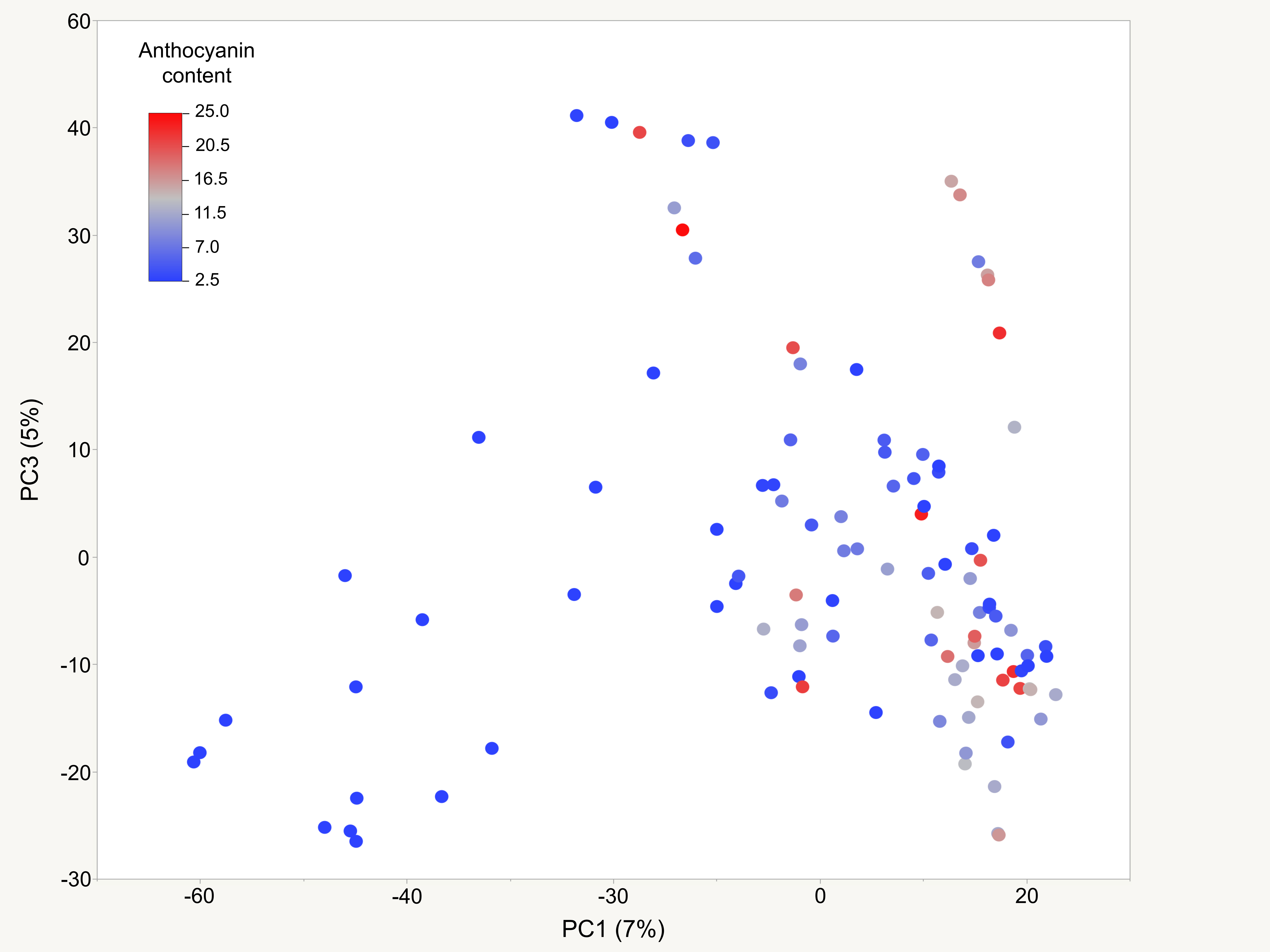

Supplement: S2 Fig — Principal components analysis of all SNPs used in the GWAS. The color of each dot indicates the total anthocyanin content (arithmetic sum of the five anthocyanins) for each plant. (PDF) [file pone.0250861.s002.pdf]
